# Correction: The effects of locomotor activity on gastrointestinal symptoms of irritable bowel syndrome among younger people: An observational study

**DOI:** 10.1371/journal.pone.0244465

**Published:** 2020-12-17

**Authors:** Toyohiro Hamaguchi, Jun Tayama, Makoto Suzuki, Naoki Nakaya, Hirokazu Takizawa, Kohei Koizumi, Yoshifumi Amano, Motoyori Kanazawa, Shin Fukudo

The affiliation for the eighth and ninth author is incorrect. Motoyori Kanazawa and Shin Fukudo are not affiliated with #3 but with #2: Department of Behavioral Medicine, Graduate School of Medicine, Tohoku University, Sendai, Japan.
