# Supplementary material for: The effects of locomotor activity on gastrointestinal symptoms of irritable bowel syndrome among younger people: An observational study
Source: PLoS One. 2020 May 29;15(5):e0234089. doi: 10.1371/journal.pone.0234089 (PMC7259724; doi:10.1371/journal.pone.0234089)
Supplement: S1 Table — Estimated probability rate for Gastrointestinal Symptoms Rating Scale (GSRS) score by ordinal logistic modeling in all participants in this study (n = 101). The Health Japan 21 recommended a daily activity level of 8500 steps/day for females and 9000 steps/day for males. IBS, irritable bowel syndrome. (DOCX) [file pone.0234089.s001.docx]

**Supporting information**

**S1 Table. Target values for daily step counts in younger people with IBS.**

| Steps/day | Probability of GSRS score relative to daily activity | | | |
| --- | --- | --- | --- | --- |
|  | 1 and 2 | 2 and 3 | 3 and 4 | 4 and 5 |
| 0 | 1.0% | 12.6% | 47.2% | 89.5% |
| 500 | 0.9% | 11.4% | 44.4% | 88.4% |
| 1000 | 0.8% | 10.4% | 41.7% | 87.2% |
| 1500 | 0.7% | 9.4% | 39.0% | 85.9% |
| 2000 | 0.7% | 8.5% | 36.4% | 84.5% |
| 2500 | 0.6% | 7.6% | 33.8% | 83.0% |
| 3000 | 0.5% | 6.9% | 31.4% | 81.3% |
| 3500 | 0.5% | 6.2% | 29.0% | 79.6% |
| 4000 | 0.4% | 5.6% | 26.8% | 77.7% |
| 4500 | 0.4% | 5.0% | 24.6% | 75.7% |
| 5000 | 0.3% | 4.5% | 22.6% | 73.6% |
| 5500 | 0.3% | 4.1% | 20.7% | 71.4% |
| 6000 | 0.3% | 3.6% | 18.9% | 69.0% |
| 6500 | 0.2% | 3.3% | 17.3% | 66.6% |
| 7000 | 0.2% | 2.9% | 15.7% | 64.1% |
| 7500 | 0.2% | 2.6% | 14.3% | 61.5% |
| 8000 | 0.2% | 2.4% | 13.0% | 58.8% |
| 8500 | 0.2% | 2.1% | 11.8% | 56.0% |
| 9000 | 0.1% | 1.9% | 10.7% | 53.3% |
| 9500 | 0.1% | 1.7% | 9.7% | 50.5% |
| 10000 | 0.1% | 1.5% | 8.7% | 47.7% |

Estimated probability rate for Gastrointestinal Symptoms Rating Scale (GSRS) score by ordinal logistic modeling in all participants in this study (n=101). The Health Japan 21 recommended a daily activity level of 8500 steps/day for females and 9000 steps/day for males.

IBS, irritable bowel syndrome
