# Supplementary material for: The effects of locomotor activity on gastrointestinal symptoms of irritable bowel syndrome among younger people: An observational study
Source: PLoS One. 2020 May 29;15(5):e0234089. doi: 10.1371/journal.pone.0234089 (PMC7259724; doi:10.1371/journal.pone.0234089)
Supplement: S2 Table — Estimated probability rate for Gastrointestinal Symptoms Rating Scale (GSRS) score by ordinal logistic modeling. The Health Japan 21 recommended a daily activity level of 8500 steps/day for females. If a female patient with IBS walked only 4000 steps/day, she will attain GSRS score 5 with probability of 78.5%, while 8500 steps/day will reduce the probability to 59.7%. IBS, irritable bowel syndrome. (DOCX) [file pone.0234089.s002.docx]

**Supporting information**

**S2 Table. Target values for daily step counts in younger females with IBS.**

| Steps/day | Probability of GSRS score relative to daily activity | | | |
| --- | --- | --- | --- | --- |
|  | 1 and 2 | 2 and 3 | 3 and 4 | 4 and 5 |
| 0 | 1.4% | 16.8% | 53.7% | 89.1% |
| 500 | 1.2% | 15.5% | 51.2% | 88.1% |
| 1000 | 1.1% | 14.2% | 48.7% | 87.0% |
| 1500 | 1.0% | 13.0% | 46.2% | 85.8% |
| 2000 | 0.9% | 11.9% | 43.7% | 84.5% |
| 2500 | 0.8% | 10.9% | 41.2% | 83.2% |
| 3000 | 0.8% | 10.0% | 38.8% | 81.7% |
| 3500 | 0.7% | 9.1% | 36.5% | 80.2% |
| 4000 | 0.6% | 8.3% | 34.2% | 78.5% |
| 4500 | 0.6% | 7.6% | 32.0% | 76.8% |
| 5000 | 0.5% | 6.9% | 29.8% | 74.9% |
| 5500 | 0.5% | 6.3% | 27.8% | 73.0% |
| 6000 | 0.4% | 5.7% | 25.8% | 71.0% |
| 6500 | 0.4% | 5.2% | 23.9% | 68.9% |
| 7000 | 0.3% | 4.7% | 22.2% | 66.7% |
| 7500 | 0.3% | 4.3% | 20.5% | 64.4% |
| 8000 | 0.3% | 3.9% | 18.9% | 62.1% |
| 8500 | 0.3% | 3.6% | 17.4% | 59.7% |
| 9000 | 0.2% | 3.2% | 16.0% | 57.3% |
| 9500 | 0.2% | 2.9% | 14.7% | 54.8% |
| 10000 | 0.2% | 2.7% | 13.5% | 52.3% |

(n=78)

Estimated probability rate for Gastrointestinal Symptoms Rating Scale (GSRS) score by ordinal logistic modeling. The Health Japan 21 recommended a daily activity level of 8500 steps/day for females. If a female patient with IBS walked only 4000 steps/day, she will attain GSRS score 5 with probability of 78.5%, while 8500 steps/day will reduce the probability to 59.7%.

IBS, irritable bowel syndrome
