# Supplementary material for: The effects of locomotor activity on gastrointestinal symptoms of irritable bowel syndrome among younger people: An observational study
Source: PLoS One. 2020 May 29;15(5):e0234089. doi: 10.1371/journal.pone.0234089 (PMC7259724; doi:10.1371/journal.pone.0234089)
Supplement: Attachment — Submitted filename: 122_plosone_Response_to_Reviewers.docx [file pone.0234089.s005.docx]

Response to Academic Editor comments

**Academic Editor comments:**

***Few things in the methods should be clarified further.***

***1. Present briefly the Rome III criteria to diagnose IBS symptoms.***

**Answer to comment 1:**

We appreciate the academic editor for the helpful comments to improve our manuscript. We added the text to explanation the Rome Ⅲ criteria in our revised manuscript as follows:

“The Rome III criteria are used to diagnose IBS symptoms, which include recurrent abdominal pain or discomfort, 3 days per month in the last 3 months (12 weeks), and are associated with two or more of the following three criteria: 1) improvement with defecation, 2) the onset is associated with a change in stool frequency, and 3) the onset is associated with a change in the stool form (appearance). To fulfil the criteria, symptom onset should occur 6 months prior to the diagnosis.” (Methods section, pages 5-6, lines 92-96)

***2. Also, describe briefly the Gastrointestinal Symptoms Rating Scale (GSRS) and how it was used in the analysis.***

**Answer to comment 2:**

We agree with you regarding this comment. We have described about the GSRS and how it was used in the analysis as follows:

“The GSRS is a disease-specific instrument of 15 items combined into 5 symptom clusters depicting reflux, abdominal pain, indigestion, diarrhea, and constipation. The GSRS has a seven-point graded Likert-type scale where “1” represents the absence of troublesome symptoms and “7” represents very troublesome symptoms.” (Methods section, page 6, lines 99-102)

“The GSRS scores were derived from the total score and divided by 15 (i.e. the 15 item subscales). The average pedometer counts (steps/day) were calculated using all days of data collection. The association between the GSRS score and pedometer counts was determined by the ordinal logistic modeling analysis [21]. The relationship between GSRS score and pedometer counts (prediction probability *g (x))* was estimated using ordinal logistic regression modeling (Equation 1) with the dependent variable as GSRS score (*f (x),* continuous variates 1 to 7) and the independent variable as pedometer counts for *x* (Equation 2). (Methods section, page 7, lines 110-116)

***3. Please describe briefly the process to obtain data from pedometer on physical activity, and how it was used in the analysis for e.g. average of all days of data or every day’s data, etc.***

**Answer to comment 3:**

We agree with you on this comment. We have described briefly the process of how to obtain data from the pedometer on the physical activity, and how it was used in the analysis as follows:

“Walking activity data that were recorded in LifeCorder GS were uploaded into a personal computer using an application Lifelyzer05 (Kenz, Tokyo).” (Methods section, page 6, lines 105-106)

“The average pedometer counts (steps/day) were calculated using all days of data collection.” (Methods section, page 7, lines 111-112)

Response to Reviewers

**Reviewer #1 comments:**

***The authors have addressed the comments in my previous review, and the manuscript appears ready to be accepted for publication. However, there are a few minor points that should be addressed as listed below:***

***1. Figure 3 is identical to Figure 2, and the image described as Figure 3 in the manuscript does not exist. This oversight is easy to remedy.***

**Answer to comment 1:**

We appreciate the reviewer’s helpful comments to improve our manuscript. We carefully checked and replaced Figures 2 and 3. The figures are very similar and at first glance they look the same, but they are different. For example, the number of scatter plots is different, and the logistic curves are slightly different. Please see the revised Figures 2 and 3.

***2. On line 87, change the word "momentum" to "activity".***

**Answer to comment 2:**

We agree with the reviewer about this comment and have changed the word "momentum" to "physical activity" for consistency with all other uses in the revised manuscript (Methods section, page 5, line 87).

***3. In lines 141 and 178, delete the word "fashion"; it is adequate without this word.***

We agree with the reviewer on this comment and have deleted the word "fashion" in the revised manuscript (Pages 9-11, lines 151-190).

***Reviewer #2: The authors addressed my question about using the Rome III criteria and revised the text and references accordingly.***

**Answer to comment:**

We would like to thank Reviewer #2 for carefully reviewing our manuscript and for the positive support of our research.
